# Genomic Variants Revealed by Invariably Missing Genotypes in Nelore Cattle

**DOI:** 10.1371/journal.pone.0136035

**Published:** 2015-08-25

**Authors:** Joaquim Manoel da Silva, Poliana Fernanda Giachetto, Luiz Otávio Campos da Silva, Leandro Carrijo Cintra, Samuel Rezende Paiva, Alexandre Rodrigues Caetano, Michel Eduardo Beleza Yamagishi

**Affiliations:** 1 Faculdade de Ciências Agrárias, Biológicas e Sociais Aplicadas, Universidade do Estado de Mato Grosso (UNEMAT), Nova Xavantina, Mato Grosso, Brazil; 2 Programa de Pós-Graduação em Genética e Biologia Molecular–Instituto de Biologia, Universidade Estadual de Campinas (UNICAMP), Campinas, São Paulo, Brazil; 3 Laboratório Multiusuário de Bioinformática (LMB)—Embrapa Informática Agropecuária, Campinas, São Paulo, Brazil; 4 Embrapa Gado de Corte, Campo Grande, Mato Grosso do Sul, Brazil; 5 Embrapa–Secretaria de Relações Internacionais, Brasília, Distrito Federal, Brazil; 6 Embrapa Recursos Genéticos e Biotecnologia, Brasília, Distrito Federal, Brazil; CSIRO, AUSTRALIA

## Abstract

High density genotyping panels have been used in a wide range of applications. From population genetics to genome-wide association studies, this technology still offers the lowest cost and the most consistent solution for generating SNP data. However, in spite of the application, part of the generated data is always discarded from final datasets based on quality control criteria used to remove unreliable markers. Some discarded data consists of markers that failed to generate genotypes, labeled as missing genotypes. A subset of missing genotypes that occur in the whole population under study may be caused by technical issues but can also be explained by the presence of genomic variations that are in the vicinity of the assayed SNP and that prevent genotyping probes from annealing. The latter case may contain relevant information because these missing genotypes might be used to identify population-specific genomic variants. In order to assess which case is more prevalent, we used Illumina HD Bovine chip genotypes from 1,709 Nelore (*Bos indicus*) samples. We found 3,200 missing genotypes among the whole population. NGS re-sequencing data from 8 sires were used to verify the presence of genomic variations within their flanking regions in 81.56% of these missing genotypes. Furthermore, we discovered 3,300 novel SNPs/Indels, 31% of which are located in genes that may affect traits of importance for the genetic improvement of cattle production.

## Introduction

Despite the strong lasting trend of decreasing costs associated with DNA sequencing caused by the continuing development of Next Generation Sequencing (NGS) technologies, SNP genotyping with DNA chips still offers the lowest cost and the most consistent solution for generating highly repeatable High-Density (HD) SNP data[1]. HD SNP genotyping panels have been made commercially available for humans and model species, as well as several agriculturally important species, such as cow [2], buffalo, goat, sheep, pig, chicken, trout [3], wheat [4], rice [5], and soybean [6], just to name a few. HD SNP data has been used in a wide range of applications, including population genetics, case-control and genome-wide association studies (GWAS), genomic evaluation and selection, and more recently copy number variation (CNV) studies [7].

In spite of the application, a portion of SNP genotyping data is always discarded from final datasets based on quality control criteria used to remove unreliable markers. A myriad of biological and technical issues can result in marker failure and low repeatability. As expected, genotyping probes cannot consistently anneal in the presence of any genomic variations (SNPs, deletions, insertions, etc) within target sequences and fail to produce accurate genotypes, or in some cases continually generate no genotypes at all, the so-called missing genotypes. Nevertheless, a recent study [8] has indicated that this issue may be more complex than previously thought because genomic variations outside target regions can prevent probes from properly annealing and performing their function as well. Thus, any genomic variation within flanking regions, even those outside probe target sequences, might hamper accurate genotyping.

The extent of the aforementioned issues is highly dependent on the divergence between populations used for probe design and the population under study. When samples are derived from the same populations used for generating sequences for probe design, this may not be an issue at all, since the odds of novel unobserved genomic variants within the same population are small. However, the usefulness of HD SNP panels relies on their ability to work on samples from diverse populations, and in these cases the aforementioned technical limitations may produce corresponding genotypes that are consistently missing in either a proportion of samples or even within the entire dataset. Most data quality control procedures routinely and indistinctly discard markers that never generate genotyping data in a specific population or breed in the same manner as other markers that produce varying low call rates. While the latter ought to be discarded because they do not contain useful or reliable information, the former should be further investigated as they might reveal population-specific genomic variant regions, where genetic divergence between populations is higher as consequence of their evolutionary past.

Contemporary bovine breeds can be subdivided into two closely related genetic groups or subspecies, which diverged 250,000 years ago [9]. Taurine (*Bos taurus*) cattle and zebuine (*Bos indicus*) cattle, were originally derived from northern Europe and the Indian continent, respectively [10], and show an average nucleotide divergence level of 117,000–275,000 B.P. [10]. The Illumina Bovine HD SNP chip was built by a multi-institutional consortium and contains a total of 777,962 polymorphic SNPs identified mostly from within-breed sequence comparisons, including data derived from taurine, zebuine and composite breeds [2]. Illumina acknowledges that sequence divergence in regions flanking assayed SNPs may potentially result in probes which are not fully compatible across all breeds, and that consequently yield lower average call rates in specific breeds when compared to most of the loci in the panel (Illumina BovineHD Genotyping BeadChip Data Sheet- http://res.illumina.com/documents/products/brochures/brochure_agriculture.pdf). Furthermore, they report that 29,968 SNPs (3.85%) which appear to be flanked by sequence polymorphisms because of breed-specific lower call rates, were retained in the HD panel because they may provide biologically relevant information (Illumina BovineHD Genotyping BeadChip Data Sheet).

An initial analysis of a dataset with genotyping data from 1,709 Nelore (zebuine) animals revealed a number of consistently missing genotypes. Do these failed SNPs observed in the Nelore breed actually reveal genomic variant? Do those hypothetical genomic variants occur within biologically relevant loci? To answer these questions, re-sequencing data from historical bulls from the breed, and automated and manual annotation of identified regions were performed.

Genotyping data from a total of 1,709 Nelore animals and re-sequenced NGS data from 8 historical sires were used to identify a total of 3,200 SNPs that consistently failed to generate genotyping data in the Nelore breed (a specific group of SNPs that will be henceforth termed SFNBs–SNPs Failed in Nelore Breed). Further investigation has shown that, within the flanking regions of these 3,200 SFNBs, there were 3,300 novel SNPs/Indels, from which 31% are located on regions containing genes. In the following sections, we present results confirming that SFNBs actually reveal divergent genomic variants between the *Bos taurus* and *Bos indicus* subspecies, and that these genomic variants observed in Nelore cattle (GVON)s can be found within genes that may affect production traits of importance for genetic improvement in cattle.

## Materials and Methods

### Animals

Specific approval from an Animal Care and Use Committee was not obtained for this study because samples had been previously collected as part of a commercial testing operation and no new animals had to be handled. The experiment was performed on genotyping data generated from DNA samples that had been previously collected. DNA was extracted from semen samples obtained from commercial companies from bulls that are in the market, and from hair and venous blood samples obtained from animals in commercial farms, as part of routine animal handling and testing procedures. Tissues were processed with standard commercial kits. The report is not intended to be a field study and none of the authors were involved in sample collection.

### SNP Genotyping and Data Analysis

A total of 1,709 Nelore samples were genotyped with the Illumina Bovine HD Genotyping BeadChip in a commercial service lab. Genotyping failure frequency was estimated for all SNP markers. Markers that failed to generate genotyping calls in all tested samples were identified and submitted to further analysis.

### NGS Data Generation and Analysis

A set of eight bulls representing historical sires in the Nelore breed were re-sequenced using Illumina HiSeq2000 100-bp paired-end reads, with an average depth coverage of >20X. Paired-end reads were mapped onto the UMD 3.1 reference bovine genome [11] through the use of Bowtie with MAQ-like alignment policy [12]. Alignment files were sorted and indexed using Samtools [13]. SNP and INDEL call procedures for each one of the 8 alignment files were performed using samtools mpileup and bcftools. No distinction was made between variations observed within Nelore sequences and between the taurine reference sequence and Nelore WGS.

Genomic variations observed within 100bp upstream and downstream (accession number at SRA: SRX973260, SRX973301, SRX973316, SRX973317, SRX973318, SRX973320, SRX973322, SRX973378) from SFNBs were identified and annotated with the Variant Effect Predictor (VEP) from Ensembl [14]. The Integrative Genomic Viewer (IGV–version 2.0.30) developed by the Broad Institute [15] was used to visualize alignment files. Distance estimates between the SNP assayed in the HD panel and the nearest observed Nelore-specific variant were calculated.

### Probe Sequences and Analysis

**Fig 1:**
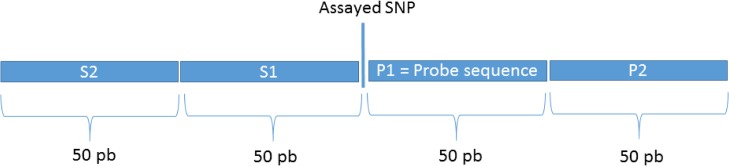
Regions defined for obtaining estimates of genomic variation. P1 represents the 50bp Illumina probe target sequence. P2 corresponds to the 50bp adjacent to P1 on the distal side of the assayed SNP. S1 and S2 are symmetrical to P1 and P2, respectively.

The complete set of the Illumina BovineHD 50bp probe sequences was downloaded from the manufacturer’s website. Each one of the 50bp probe sequences was blasted against the UMD3.1 reference bovine genome. This procedure was necessary for the acquisition of both the probes’ genomic start and end positions and their strand orientation. A C++ program was developed to integrate all the aforementioned information and to classify observed genomic variations according to their position in relation to each SFNB: 50bp Illumina probe target sequence (P1), 50bp adjacent to P1 on the distal side of the assayed SNP, and the symmetrical regions to P1 (S1) and P2 (S2) (see Fig 1).

### Functional Annotation of SNP-Containing Genes

Fasta sequences of genes containing at least one identified SFNB were imported into Blast2GO [16] (http://www.blast2go.de/) for automated functional annotation. The dataset was blasted against NCBI nr database with default parameters (with an e-value threshold of 1e-03 and an HSP length cut-off of 100) using blastx. Mapping of sequences to GO terms and GO term assignments were performed using default parameters (an e-value hit filter of 1e-06, annotation cut-off of 55 and a GO weight of 5). Annotations were further augmented using the Annex function of the GO Annotation Toolbox [17]. InterProScan terms were obtained [18] and Kegg pathway maps (http://www.genome.jp/kegg/pathway.html) were downloaded for all enzyme codes. The same procedure was adopted for the automatic functional annotation of genes with identified synonymous substitutions in flanking regions of assayed SNPs.

## Results

**Fig 2:**
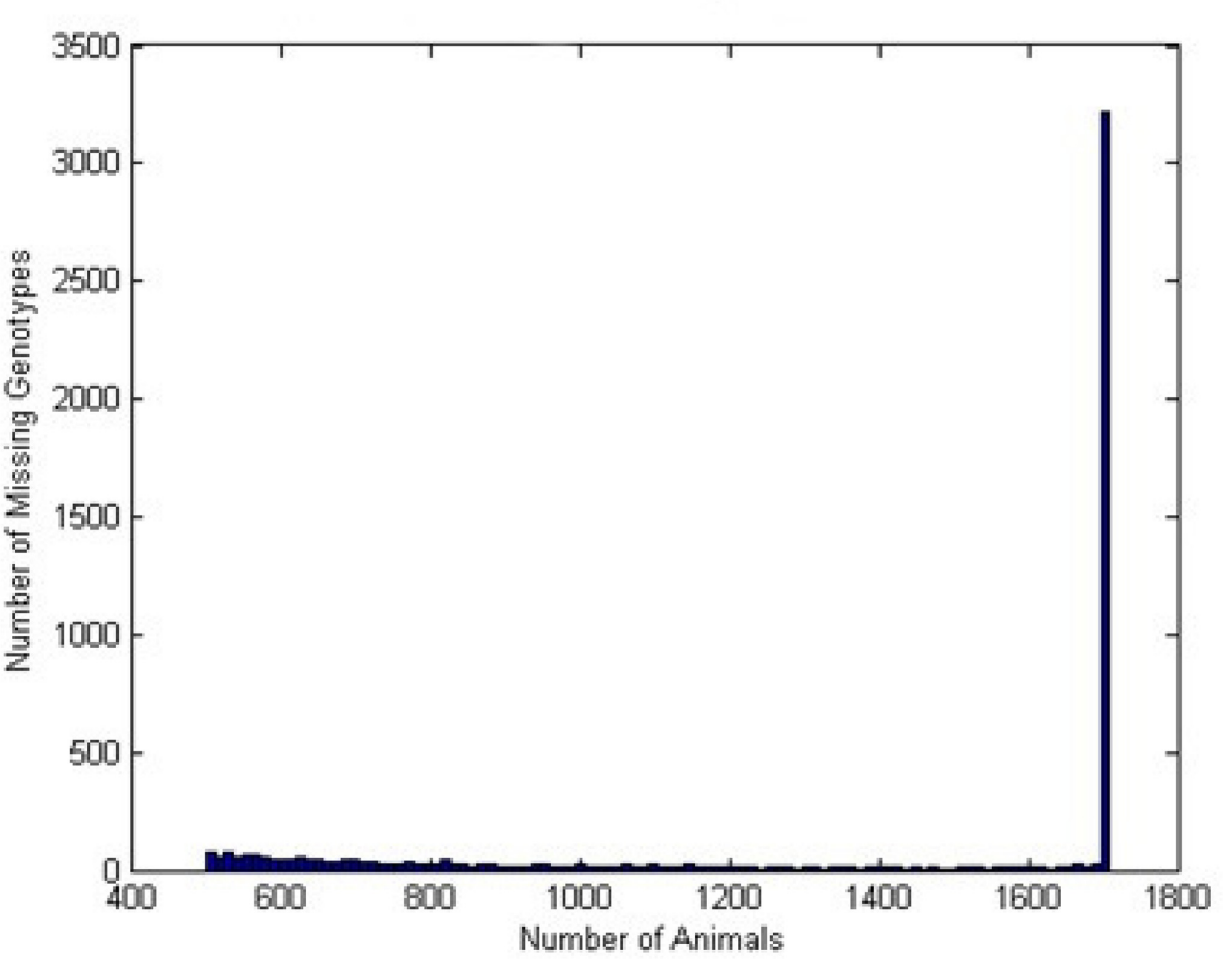
Frequency of missing genotypes in Nelore cattle in a total of 1,709 samples tested with the Illumina Bovine HD.

**Fig 3:**
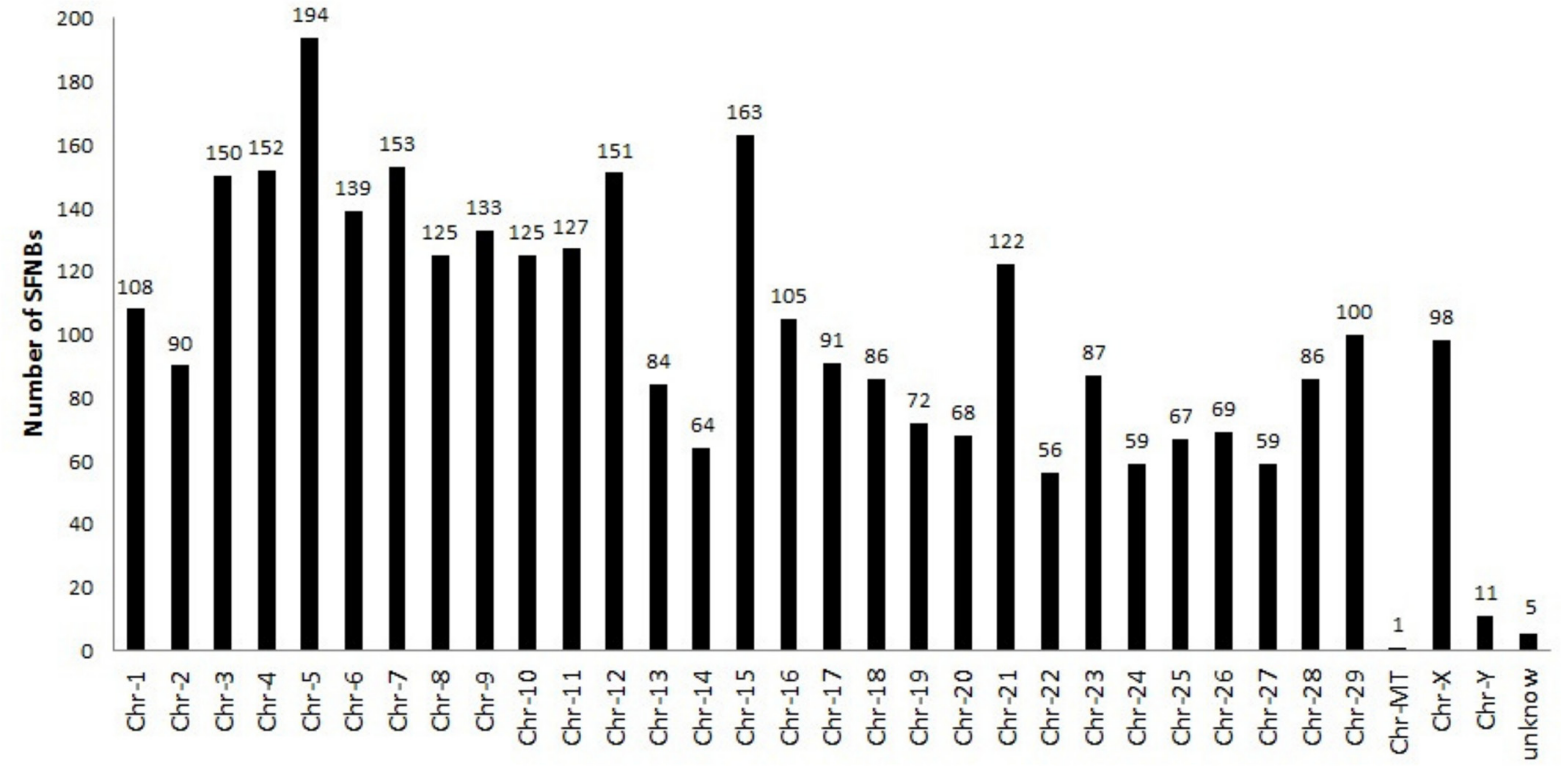
Distribution of SFNBs across bovine chromosomes.

A total of 3,200 SFNBs were identified in all of the 1,709 Nelore samples evaluated (Fig 2). The number of SNPs observed to be missing in only part of the genotyped samples was minimal. The number of observed SFNBs was not found to be evenly distributed across chromosomes (Fig 3), and the correlation with chromosome size was estimated to be 0.58. Mean concordance observed between genotype calls obtained from the Bovine HD BeadChip and WGS data from eight animals was 99.5%.

**Fig 4:**
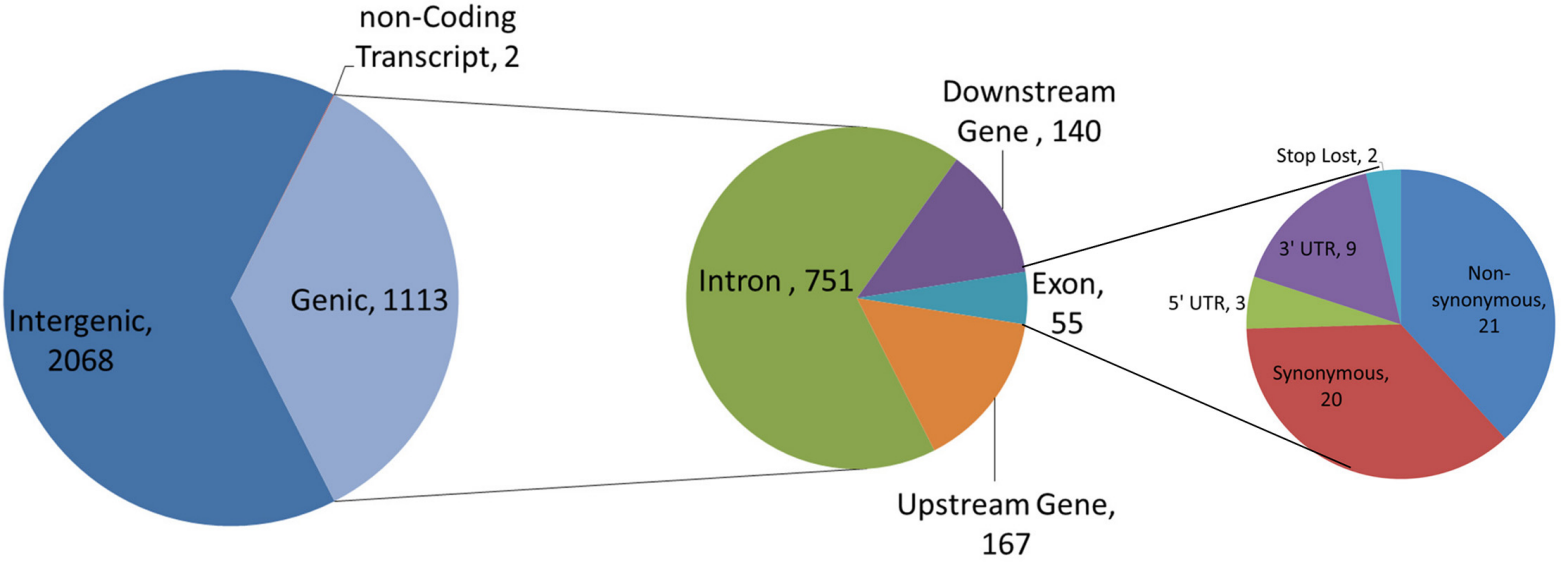
Functional characterization of 3,183 SNP markers derived from the Illumina Bovine HD panel that consistently generated missing genotypes in the Nelore breed (SFNBs).

Fig 4 summarizes the functional analysis performed with 3,183 SFNBs (17 SFNBs are located on mtDNA, Y-specific regions or unmapped chromosomes and were not considered in the subsequent analyses—see S1 Table). The analysis revealed that 2,068 SNPs (64.97%) are located within intergenic regions (Fig 4) while 1,113 SNPs are located in intragenic regions: 751 SNPs (23.59%) are located within introns, 167 (5.25%) are upstream and 140 (4.4%) are downstream of assayed SNPs, 21 (0.66%) are non-synonymous variants, 20 (0.63%) are synonymous variants, 9 (0.28%) are located on 3’ UTR regions, 3 (0.09%) are located on 5’ UTR regions, 2 (0.06%) result in stop loss variants and 2 (0.06%) were found to be located on non-coding transcripts.

**Fig 5:**
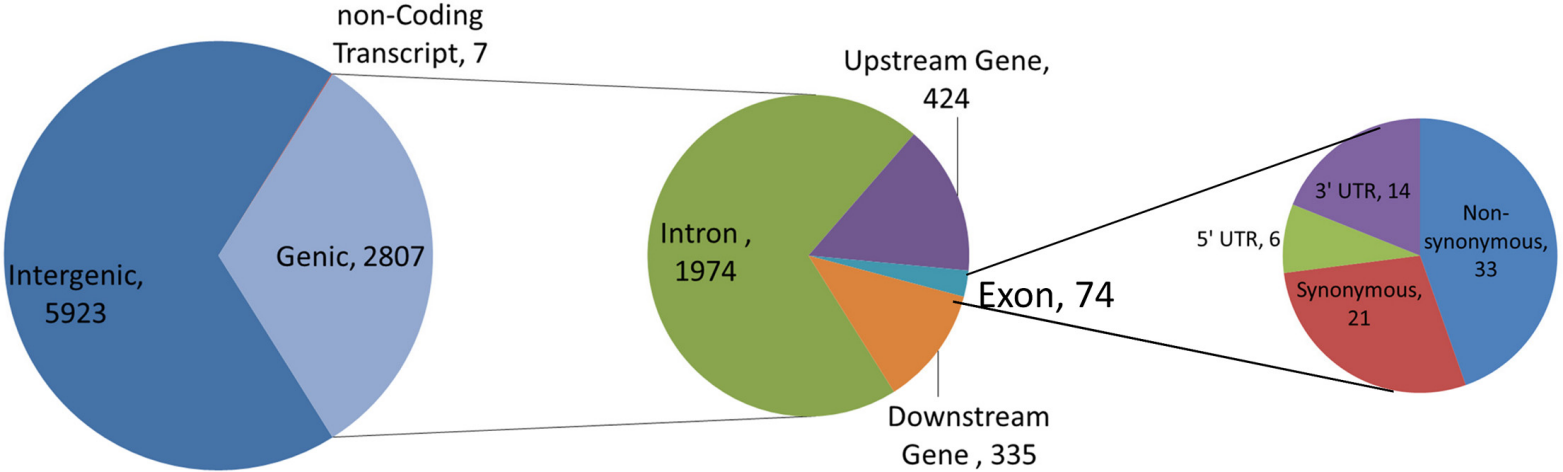
Functional characterization of 8,837 SNPs and INDELs identified within 100bp regions flanking SNPs assayed in the HD panel.

The SNP call procedure on flanking regions around assayed SNPs (Fig 1) revealed 8,840 SNPs/INDELs, 3,300 of which are novel (see S2 Table). A total of 8,737 SNPs were annotated with VEP. A total of 2,807 (32.12%) SNPs were found within intragenic sequences. From these, 1,974 SNPs are located on introns, 424 and 335 SNPs are up and downstream from coding sequences, respectively, and 74 SNPs are located on exons (Fig 5). A total of 14 SNPs were observed within 3’UTRs and 6 SNPs within 5’UTR. Twenty-one synonymous substitutions and 32 non-synonymous substitutions were observed in 20 different genes (Fig 5).

**Fig 6:**
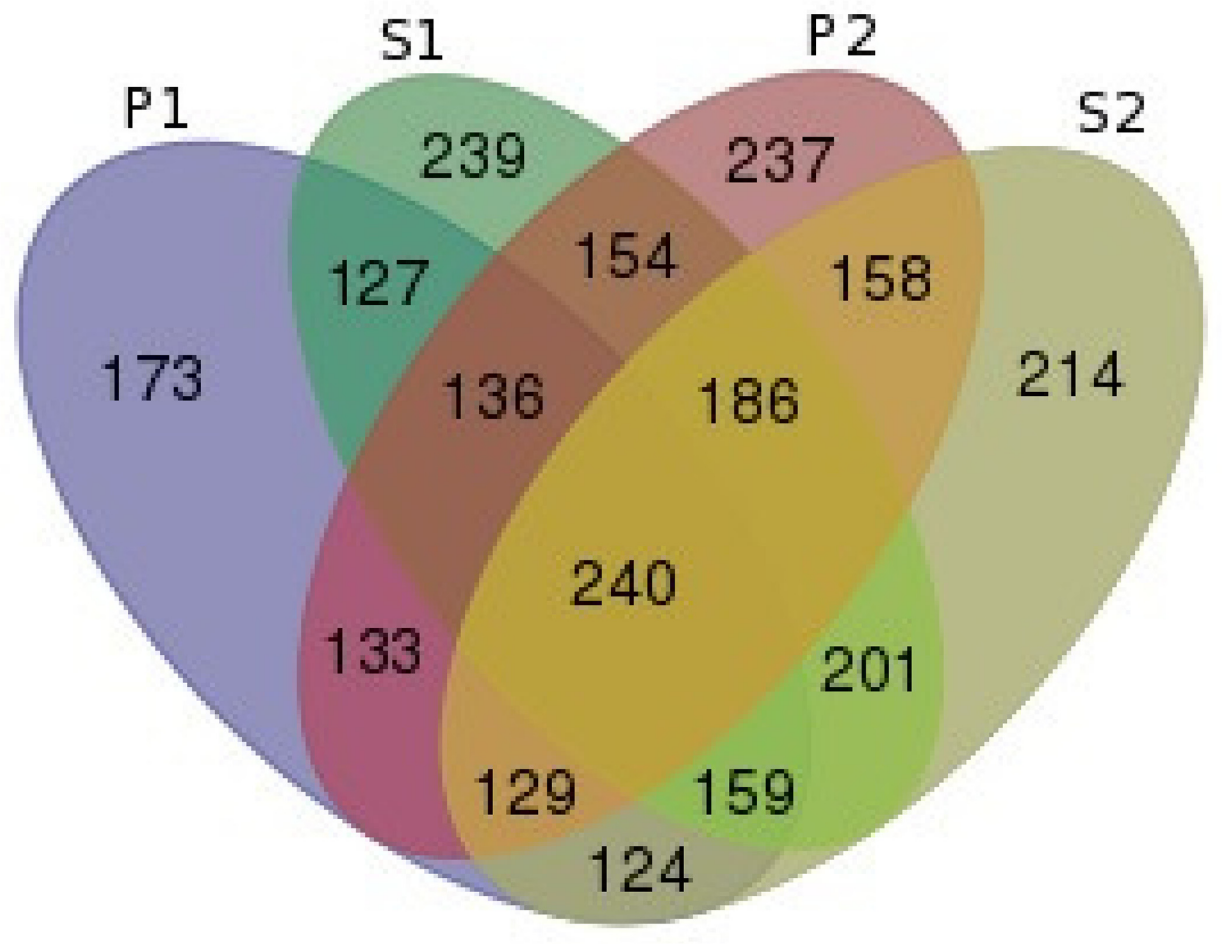
Number of non-redundant SFNBs in regions flanking SNPs assayed in the Illumina Bovine HD panel (see S1 Table).

Fig 6 shows the number of non-redundant SFNBs across the P1, S1, P2, and S2 regions (see S1 Table). Novel SNPs/INDELs were observed in the vicinity of 2,610 SFNBs (81.56%). Further classification of these SNPs revealed that at least one novel SNP was observed in the P1 region of 1,221 assayed SNPs, while 1,442, 1,373 and 1,441 SNPs were observed in the S1, P2, and P3 regions, respectively. Variants were observed within all four regions in 240 assayed SNPs.

**Fig 7:**
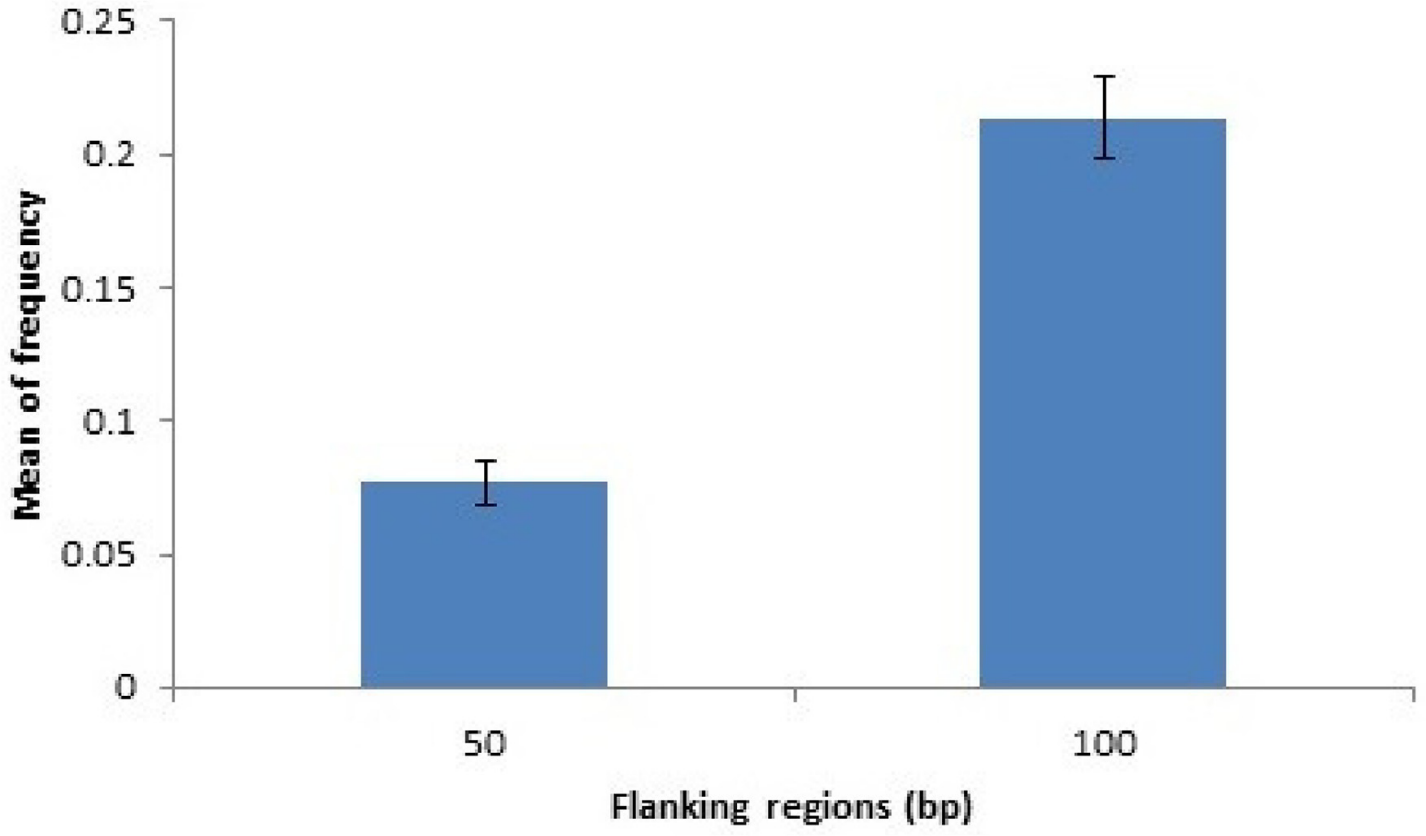
Mean frequency and standard deviation of the nearest Nelore SNPs within 50bp and 100bp size bins.

Distance estimates between assayed SNPs and the nearest novel Nelore SNP/INDEL observed in the resequencing data are shown in Fig 7. Variants were observed within 50bp and 100bp of the HD Illumina assayed SNP in a total of 7.68% and 21.32%, respectively.

## Discussion

The distribution of the HD Illumina SNPs within bovine chromosomes is proportional to chromosome size. If the chromosomal distribution of the SFNBs were random, we would expect that larger chromosomes would contain higher numbers of SFNBs, but that was not observed (Fig 3). In fact, BTA5 was found to have the highest number of SFNBs (n = 194), followed by BTA15 (n = 163), BTA7 (n = 153), BTA4 (n = 152), BTA12 (n = 151), and BTA3 (n = 150). In a recent study in which the same HD genotyping chip was used to search for divergent regions between zebuine and taurine cattle [19], the authors reported large regions comprised of millions of base pairs, on BTA 3, 4, 5, 7, and 12. The divergent regions were ranked in the top 1% for values of loci under positive selection. Even though BTA1 represents the largest chromosome in the bovine genome, it is absent from both lists. BTA15 was identified in our list but not in the previous study. The described methodology only included SNPs with more than 95% successful genotypes, and therefore we are led to conclude that all SFNBs were discarded from this study [19]. Additional genomic regions divergent between taurine and zebuine cattle have also been reported on BTA 3, 4, 5, 7, 12, and 15 [20]. Even though three distinct strategies were used in [19], [20] and the present report, the same chromosomes were identified to contain divergent regions between taurine and zebuine cattle, reinforcing that complementary results can be obtained with different methods. The use of missing genotypes in our analysis captured fine-grained information overlooked by traditional selection signature methods.

SFNBs could result from hybridization problems caused by technical issues on the chip and/or genotyping probes, rather than the presence of genomic variations within flanking regions. In these cases specific markers should always fail, in whichever breed or population tested. To test this possibility, we used HD Illumina genotypes from 52 animals (http://www.animalgenome.org/repository/cattle/Illinoi_Beever_Project.2012/) from different cattle breeds (Angus, Simmental and crossbreds) and confirmed that 3,019 out of the 3,200 SFNBs worked in most samples tested (see S3 Table). Moreover, this confounding factor was minimized even more in the current study by using NGS re-sequencing data to identify sequence variations within the vicinity of each selected locus that could explain the hybridization failure. At least one GVON was observed within 100bp in 81.56% of SFNBs, which could directly or indirectly [8] affect binding of genotyping probes. NGS resequencing data revealed GVONs 100bp up or downstream in only 21.32% of the Illumina Bovine HD SNPs. Therefore, the probability of observing a variant in the Nelore breed within an SFNB is almost four times higher than that of any other SNP in the Illumina HD panel. The odds are higher still when the region is reduced to less than 50bp. GVONs were observed within 62,53% of the 3,200 SFNBs when the P1 and S1 regions were considered. Furthermore, GVONs were observed within 50bp of the assayed SNPs in the Illumina HD panel in only 7.68% of cases. Therefore, it can be concluded that the presence of a GVON within 50bp of a SNP in the Illumina HD panel is eight times more likely to occur when we consider one of the 3,200 SFNBs. Thus, SFNBs can be considered good indicators of genomic regions containing variants between *Bos taurus* and *Bos indicus* subspecies. Genotyping failure in 18.44% of SFNBs could not be explained by SNP or INDEL variants within 100bp up or downstream of the respective SNP´s. Genotyping failure was also observed in other tested breeds (S3 Table) in a total of 59 of these SNPs, suggesting technical issues in probe manufacturing may be the cause for observed missing genotypes. The remaining 531 SFNBs may have been caused by other types of genomic variations further away from assayed SNPs which could not be elucidated with the analyzed data.

**Fig 8:**
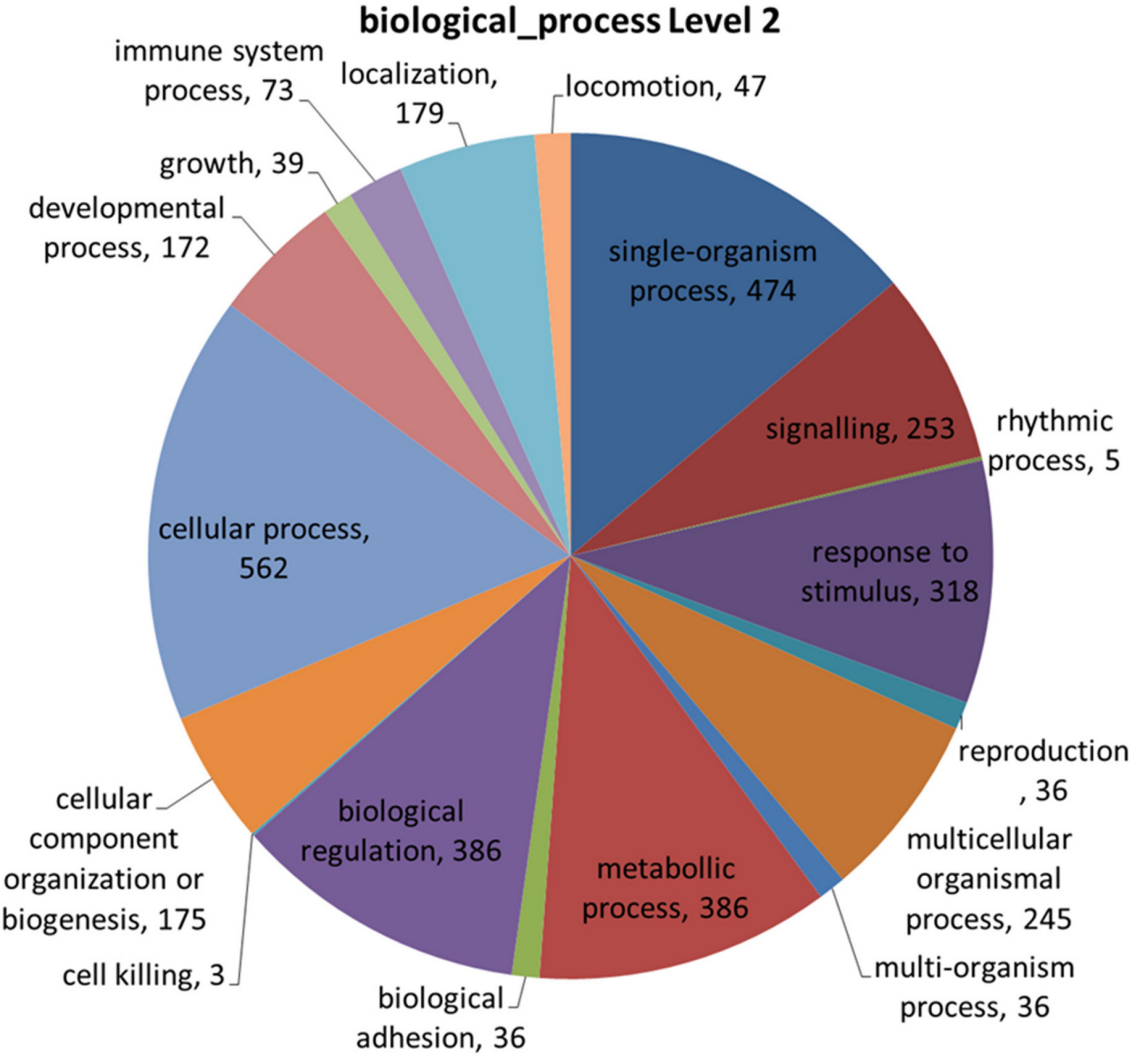
GO annotation of biological processes affected by genes that were identified by SFNBs from the Illumina Bovine HD panel.

GO annotation of SFNB-containing genes revealed several categories, including biological regulation, response to stimuli, signaling, immune system processes, growth, and reproduction (Fig 8). Genes involved in these biological processes are responsible for phenotypic differences that have already been described between taurine and zebuine cattle and which are target traits in breeding programs, such as reproductive function (age of puberty, estrous cycle patterns and behavior, ovulatory capacity, reproductive hormone levels, mean number of preantral follicles) [21], resistance to endo- and ecto-parasites [22], response to heat-stress [23], susceptibility to bovine spongiform encephalopathy [24], and growth, carcass, and meat quality traits [25]. Among the SFNB-containing genes found (S2 Table), some noteworthy genes include PPARG (peroxisome proliferator-activated receptor gamma), which is the main regulator of adipogenesis and which is involved in intramuscular fat deposition (marbling) [26–30] and has been associated with age of puberty [31] in cattle. The genes found also included CAST genes (calpastatins) and calpain (CAPN) inhibitors, which are both accountable for post-mortem muscle fiber proteolysis and associated with shear force and tenderness in the skeletal muscles [32, 33].

**Fig 9:**
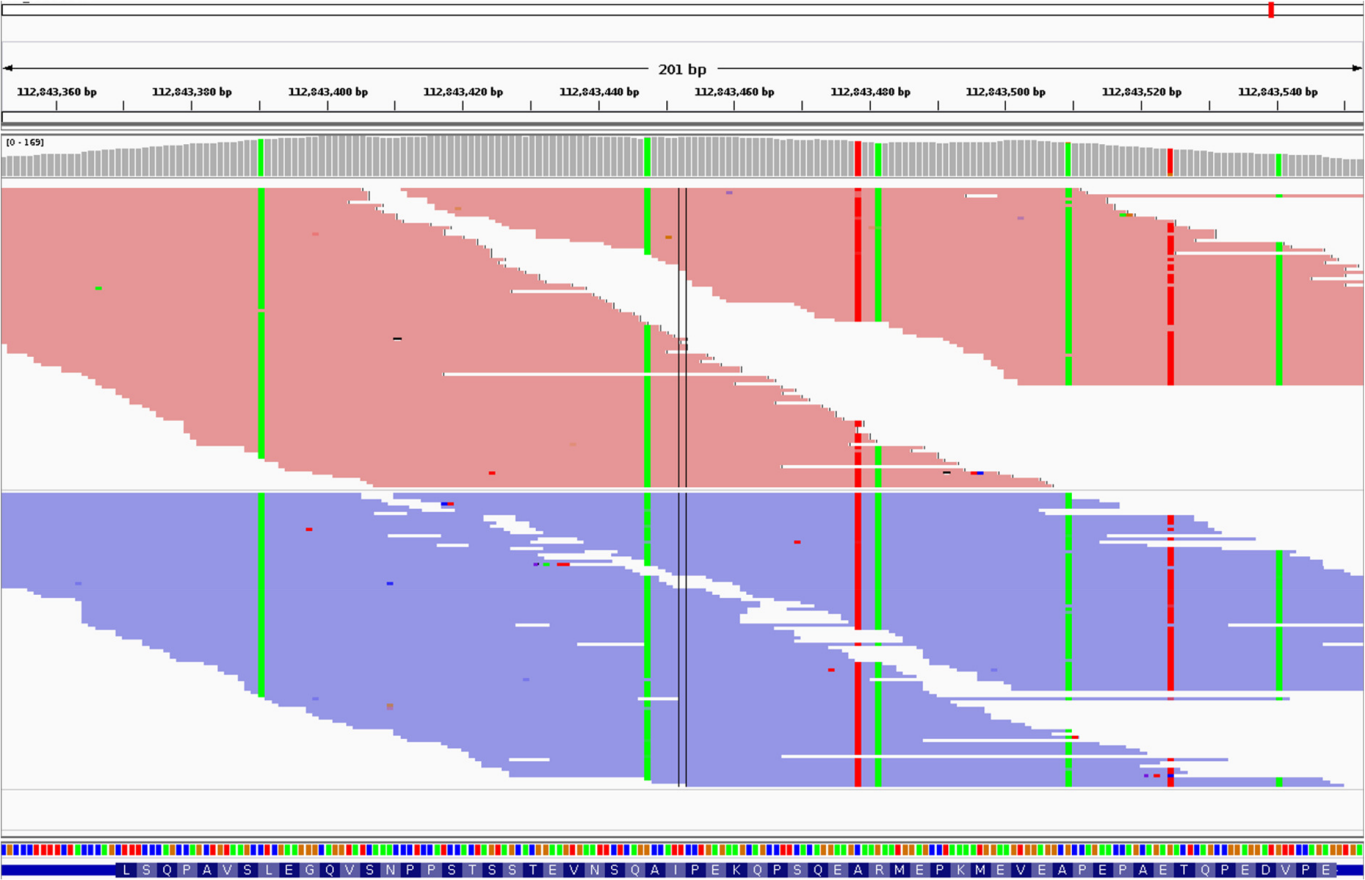
IGV screenshot image. The double vertical lines indicate the BovineHD0500032585 SNP position. Colored positions indicate flanking SNPs. There are 6 non-synonymous SNPs and 1 synonymous SNP (4^th^ column from left).

Major histocompatibility complex (MHC) class I- (MR1) and class II-related genes (BOLA-DRB3, BOLA-DQA1, BOLA-DQA2), which are central to immunity and are among the most polymorphic genes known [34], were also found. Other SFNB-containing genes involved in the immune system that were identified include T-cell receptors, a TCR-α chain (which reacts with antigenic protein peptides in the context of self major histocompatibility complex (MHC) proteins), and a TCR-γ chain (which reacts with proteins that do not involve MHC presentation) [35], and CD6, a T-cell surface protein that regulates antigen-specific responses through cell-cell contact [36]. Considering the 8,737 SNPs identified in SFNB flanking regions annotated with VEP, 32 SNPs out of the 74 SNPs that were found to be located within exons resulted in non-synonymous substitutions (Table 1). An extreme case of non-synonymous mutation is shown in Fig 9. In the flanking regions of the BovineHD0500032585 SNP, there are 7 interspecies mutations, 6 of which are non-synonymous and only 1 of which is synonymous. The BovineHD0500032585 SNP is located on BTA5 at position 112,843,452 bp within an exon of EP300 (Table 1). According to Gayther et al. [37], EP300 regulates transcription through chromatin remodeling and plays a major role in cell proliferation and differentiation processes. Furthermore, in cattle, this gene has been associated with lipid metabolism [38], which is important in beef cattle meat quality. Another extreme case of non-synonymous mutations was observed in the flanking regions of BovineHD0100043813: there are 4 non-synonymous SNPs within an exon of the RIPPLY3 gene. The literature on this gene is scarce, but a recent study has shown that it is a repressor of the Tbx1 gene, which plays a major role in morphogenesis. It is also required for the development of the pharyngeal apparatus in mice [39], which is essential for eating and respiration.

**Table 1:** Detailed information for 32 SNPs located within coding regions flanking SFNBs which resulted in non-synonymous substitutions. An extreme case of non-synonymous mutations in gene EP300. Large numbers of olfactory receptor genes were found to contain SNPs that result in non-synonymous substitutions.

HD Illumina SNP	SNP Position	Neighbor SNP Position	Allele	Ensembl Gene ID	Ensembl Transcript ID	Associated Gene Name	Description	Amino acids	Codons	Strand
BovineHD0100043813	1:150972066	1:150972082	T	ENSBTAG00000036019	ENSBTAT00000050509	RIPPLY3	ripply transcriptional repressor 3	R/L	cGc/cTc	1
BovineHD0100043813	1:150972066	1:150972082	T	ENSBTAG00000036019	ENSBTAT00000050510	RIPPLY3	ripply transcriptional repressor 3	R/L	cGc/cTc	1
BovineHD0100043813	1:150972066	1:150972144	T	ENSBTAG00000036019	ENSBTAT00000050510	RIPPLY3	ripply transcriptional repressor 3	R/W	Cgg/Tgg	1
BovineHD0100043813	1:150972066	1:150972145	A	ENSBTAG00000036019	ENSBTAT00000050510	RIPPLY3	ripply transcriptional repressor 3	R/Q	cGg/cAg	1
BovineHD0100043813	1:150972066	1:150972159	A	ENSBTAG00000036019	ENSBTAT00000050510	RIPPLY3	ripply transcriptional repressor 3	D/N	Gat/Aat	1
BovineHD0300002957	3:8944823	3:8944722	T	ENSBTAG00000024849	ENSBTAT00000007838	LOC100138271	oviduct-specific glycoprotein-like	T/I	aCt/aTt	1
BovineHD0500032585	5:112843452	5:112843390	A	ENSBTAG00000016198	ENSBTAT00000021556	EP300	EP300 interacting inhibitor of differentiation	L/I	Ctc/Atc	1
BovineHD0500032585	5:112843452	5:112843447	A	ENSBTAG00000016198	ENSBTAT00000021556	EP300	EP300 interacting inhibitor of differentiation	A/T	Gcc/Acc	1
BovineHD0500032585	5:112843452	5:112843478	T	ENSBTAG00000016198	ENSBTAT00000021556	EP300	EP300 interacting inhibitor of differentiation	A/V	gCa/gTa	1
BovineHD0500032585	5:112843452	5:112843481	A	ENSBTAG00000016198	ENSBTAT00000021556	EP300	EP300 interacting inhibitor of differentiation	R/K	aGa/aAa	1
BovineHD0500032585	5:112843452	5:112843524	T	ENSBTAG00000016198	ENSBTAT00000021556	EP300	EP300 interacting inhibitor of differentiation	E/D	gaG/gaT	1
BovineHD0500032585	5:112843452	5:112843540	A	ENSBTAG00000016198	ENSBTAT00000021556	EP300	EP300 interacting inhibitor of differentiation	V/I	Gtt/Att	1
BovineHD0700005952	7:21542670	7:21542589	A	ENSBTAG00000010373	ENSBTAT00000013697	CACTIN	cactin, spliceosome C complex subunit	R/Q	cGg/cAg	1
BovineHD1100012858	11:44163513	11:44163632	C	ENSBTAG00000022461	ENSBTAT00000056779	SEPT10	septin 10	M/T	aTg/aCg	1
BovineHD1100027304	11:93678380	11:93678280	C	ENSBTAG00000045527	ENSBTAT00000063061	LOC539172	olfactory receptor, family 1, subfamily J, member 4-like	K/R	aAg/aGg	-1
BovineHD1200020012	12:72533573	12:72533699	C	ENSBTAG00000047181	ENSBTAT00000035476	LOC530803	aTP-binding cassette, sub-family C (CFTR/MRP), member 4-like	K/E	Aag/Gag	-1
BovineHD1200020012	12:72533573	12:72533707	G	ENSBTAG00000047181	ENSBTAT00000035476	LOC530803	aTP-binding cassette, sub-family C (CFTR/MRP), member 4-like	L/P	cTg/cCg	-1
BovineHD1300021565	13:74675560	13:74675685	C	ENSBTAG00000039693	ENSBTAT00000066021	LOC618696	BPTI/Kunitz family of serine protease inhibitors	I/T	aTt/aCt	1
BovineHD1500014805	15:51516836	15:51516834	C	ENSBTAG00000015357	ENSBTAT00000028579	OR52B4	olfactory receptor, family 52, subfamily B, member 4	H/R	cAt/cGt	-1
BovineHD1700000025	17:59578	17:59498	A	ENSBTAG00000048129	ENSBTAT00000064742	OR5I1 (Similar)	olfactory receptor, family 5, subfamily I, member 1	S/F	tCt/tTt	-1
BovineHD1700000025	17:59578	17:59511	T	ENSBTAG00000048129	ENSBTAT00000064742	OR5I1 (Similar)	olfactory receptor, family 5, subfamily I, member 1	E/K	Gag/Aag	-1
BovineHD1700000025	17:59578	17:59600	A	ENSBTAG00000048129	ENSBTAT00000064742	OR5I1 (Similar)	olfactory receptor, family 5, subfamily I, member 1	S/L	tCa/tTa	-1
BovineHD1800015304	18:52121851	18:52121877	A	ENSBTAG00000001260	ENSBTAT00000031940	PINLYP	Phospholipase A2 inhibitor and LY6/PLAUR domain containing	V/M	Gtg/Atg	1
BovineHD1900002770	19:9851703	19:9851769	A	ENSBTAG00000003280	ENSBTAT00000004247	MGC137055	uncharacterized protein MGC137055	V/I	Gtc/Atc	1
BovineHD1900015325	19:54647984	19:54647899	A	ENSBTAG00000018661	ENSBTAT00000042576	TMC6	transmembrane channel-like 6	P/H	cCt/cAt	1
BovineHD2000016716	20:59531547	20:59531546	A	ENSBTAG00000021972	ENSBTAT00000061278	DNAH5	dynein, axonemal, heavy chain 5	V/M	Gtg/Atg	1
BovineHD2100005821	21:20129619	21:20129578	C	ENSBTAG00000045929	ENSBTAT00000036189	LOC512440	myeloid-associated differentiation marker-like	M/V	Atg/Gtg	-1
BovineHD2300007434	23:27155608	23:27155565	G	ENSBTAG00000006864	ENSBTAT00000045468	LOC781663	complement C4-A-like	S/G	Agc/Ggc	1
BovineHD2300008405	23:29518476	23:29518485	T	ENSBTAG00000038562	ENSBTAT00000054810	LOC784787	olfactory receptor, family 10, subfamily C, member 1-like	A/V	gCt/gTt	1
BovineHD2300015228	23:52445428	23:52445402	G	ENSBTAG00000047485	ENSBTAT00000064488	OR5M10	olfactory receptor, family 5, subfamily M, member 10	V/L	Gtg/Ctg	-1
BovineHD2600015134	26:16287928	26:16287953	C	ENSBTAG00000023955	ENSBTAT00000007005	LOC505468	cytochrome P450 family 2 subfamily C polypeptide 18-like	V/A	gTg/gCg	1
BovineHD2900011969	29:39707691	29:39707754	C	ENSBTAG00000046686	ENSBTAT00000063468	LOC616004	pregnancy-associated glycoprotein 1-like	E/G	gAa/gGa	-1

A large number of olfactory receptor genes (OR) was found to contain SNPs that result in non-synonymous substitutions as well (Table 1). Vertebrate olfactory receptors (OR) are G-protein linked transmembrane receptors that constitute the largest superfamily in the mammalian genome [40], with genes located in genomic clusters dispersed over different chromosomes [41]. In the animal kingdom, the sense of smell plays a major role in survival and reproduction. For this reason, animals need to detect and discriminate a large number of chemical compounds [42]. In mammalian evolution, in a change that was likely due to the need to adapt to different environments, the number of OR genes varies widely [43]. As reviewed by Iskow et al. [44], many CNVs in humans include genes or gene families that may have been under positive selection and which also allow for the adaptation to new environments and challenges. Recent CNV studies in cattle revealed a large number of genes from the OR family in these regions [45–53]. The OR gene repertoire in cattle was identified and analyzed by Lee et al. [41]. The authors suggest that the study of OR variation within species is likely to reveal important biological information associated with traits of for determining the economic importance for livestock production. A non-synonymous mutation flanking BovineHD2000016716 was also observed within a gene affecting the respiratory system. DNAH5 is associated with the onset of Primary Ciliary Dyskinesia (PCD), a respiratory disease characterized by recurrent infections of the respiratory tract and sperm immobility [54].

Our study has shown that often-discarded missing genotypes can be effectively used to identify population-specific genomic variants which in turn can be used in a wide range of applications. Although whole-genome shotgun sequences can be used to identify the underlying mutations associated with missing genotypes, more cost-effective approaches based on targeted re-sequencing could be used more efficiently, minimizing demands for complex bioinformatics procedures. Recent studies comparing genotyping data from different tissues from the same individual have shown compelling evidence that it is possible to observe tissue-dependent genotypes [55–59]. In this regard, HD genotyping data allows for not only the identification of discordant tissue-dependent genotypes, but also the discovery of new genomic variants as well. We acknowledge that only those variant loci near known SNPs can be discovered, which is a non-negligible weakness. This implies that the chances of success in finding new genomic variants rise as the number of genotyping probes within the chip increases. Companies that manufacture genotyping chips could develop denser HD genotyping chips and minimize this weakness by designing probes to cover every non-repetitive loci in the genome under study. This prospect is a trend at least in humans as the CytoScanHD Human array from Affymetrix has 2.67 million probes, 1.9 of which are non-polymorphic and designed to empower the results of CNV studies, but which are also compatible with our approach. Thus, the odds of success are therefore higher for the human model, since it has the heaviest density of any SNP panel currently available. The majority of most frequent genomic variants has already been identified in humans however, underlying mutations such as those found in the rare genetic diseases or harmful somatic mutations are likely to be rare. Missing genotype data could be used as a complementary approach to search for these mutations, as discussed in the following paragraphs.

In human case-control studies HD genotyping data is usually used to identify genotypes or genomic regions associated with a given disease considering two clear premises: (i) patients (cases) were necessarily born with the affected/susceptible genotype; and (ii) the associated genetic marker(s) must be assayed on the HD genotyping chip, or at least be in linkage disequilibrium (LD) with a SNP that is. Most hereditary diseases satisfy the first premise, and the latter is likely to hold true because the most frequent human polymorphisms have been uncovered by the NGS re-sequencing of thousands of samples from different populations [60, 61]. Therefore, it is more likely that causative mutations will be in LD with SNPs in the HD panels, rather than actually being the SNP on the HD panels. In these cases, the best result that classical approaches can initially deliver is a large genomic region associated with the disease. If the objective is to actually find the causative mutation, then the best way to do so is arguably to re-sequence some affected individuals [62]. Because of the sheer number of rare genetic diseases, however, this is not always an affordable option [63]. In cases in which the position of causative mutations are unknown, and considering the fact that the HD genotyping data of some individual cases are already available, we strongly recommend the use of missing genotype data as a complementary method to identify associated genomic variants. If by chance the causative mutation is within flanking regions of an assayed SNP, it should be identified. Clearly, the best candidate variants would be those present in all affected individuals and not present in the controls. This simple filtering strategy and some additional biological knowledge on the disease should be sufficient for reducing the number of candidate markers for further investigation.

In addition to heritable disease-causing mutations, random or induced DNA alterations may appear in somatic cells after birth and may result in severe illness, such as some cancer types [64, 65]. In these cases, the “causative mutation” need not be one mutation but can be represented by several mutations [66]. Sometimes, the knowledge of the most common and consistent variant loci may provide some insight into the diagnostic test, or even a possible treatment. With minor adaptations, our strategy could be used to determine the most frequent mutations. The adaptations that are required by the new premises are as follows: (i) the mutated genotypes appeared after birth; and (ii) there are several mutated loci. From the first premise, instead of N controls and N cases, it is only necessary to have N cases; for the second, the scope of the search should include a set of recurring mutations. From the knowledge of the disease, it should be possible to isolate normal tissues from affected ones. Thus, each individual will actually be simultaneously a case and a control through its contribution of both normal (actual birth genotype) and affected tissue (acquired mutations) samples. This strategy has already been used with NGS data [67, 68], but it is relatively expensive. High costs negatively affects the number of samples tested, and the strategy requires complex and time-consuming bioinformatics analyses. If the disease is caused by the same set of mutations, every descending affected tissue sample will consequently have them, even though additional new mutations will likely be acquired subsequently. Unlike NGS sequencing technologies, through which these last spurious mutations will result in high noise, these spurious mutations are invisible in genotyping technologies. They should be much less frequent than the primary mutations, and unaffected cells would deliver non-mutant DNA that would certainly hybridize to assay probes. Thus, only the frequent mutations are detected through this genotyping technique. This is an advantage when the ultimate goal is to identify genomic variants present in all affected samples both from the same individual and among various individuals. To reduce the number of candidate loci, the first filter should exclude all missing genotypes present in both normal and affected samples, because they most likely reflect population divergences or technical problems in the chip and therefore cannot be taken as disease-related mutations. The remaining loci may be viewed as a putative “disease mutation map,” or the most frequent variant loci that should be investigated further.

Missing genotypes have been predominantly considered an issue to be addressed through imputation-like methods [69, 70]. Only a handful of studies recognized that they could carry relevant indirect information such as the identification of deletion polymorphisms [71, 72]. These latest approaches resemble ours, since they actually use missing genotypes instead of discarding them, but do not necessarily harness all of the potential information that missing genotypes could provide. To the best of our knowledge, our work is the first to successfully show this potential and to demonstrate that missing genotypes could indeed have significant value.

## Supporting Information

S1 Table Complete list of SFNBs identified in all of the 1,709 Nelore samples. (XLSX)

S2 Table Complete list of SNPs/INDELs flanking SFNBs identified in resequencing data. (XLSX)

S3 Table Genotypes from 52 animals from different cattle breeds (Angus, Simmental and crossbreds) and confirmed that 3,019 out of the 3,200 SFNBs. (XLSX)
